# Multi-mechanism collaboration enhanced photoacoustic analyzer for trace H_2_S detection

**DOI:** 10.1016/j.pacs.2023.100449

**Published:** 2023-01-04

**Authors:** Min Guo, Xinyu Zhao, Ke Chen, Dongyu Cui, Guangyin Zhang, Chenxi Li, Zhenfeng Gong, Qingxu Yu

**Affiliations:** School of Optoelectronic Engineering and Instrumentation Science, Dalian University of Technology, Dalian 116024, Liaoning, China

**Keywords:** Multi-mechanism collaboration enhanced, Fiber-optic silicon cantilever acoustic sensor, Photoacoustic spectroscopy, SF_6_ background, Trace H_2_S analyzer

## Abstract

To realize the real-time highly sensitive detection of SF_6_ decomposition product H_2_S, a multi-mechanism collaboration enhancement photoacoustic spectroscopy analyzer (MCEPA) based on acoustic resonance enhancement, cantilever enhancement and excitation light enhancement is proposed. An SF_6_ background gas-induced photoacoustic cell (PAC) was used for acoustic resonance (AR) enhancement of the photoacoustic signals. A fiber-optic acoustic sensor based on a silicon cantilever is optimized and fabricated. The narrow-band acoustic signal enhancement based on cantilever mechanical resonance (MR) is realized in the optimal working frequency band of the PAC. A fiber-coupled DFB cascaded an Erbium-doped fiber amplifier (EDFA) realized the light power enhancement (LPE) of the photoacoustic signals excitation source. Experimental results show that the MR of the fiber-optic silicon cantilever acoustic sensor (FSCAS) is matched with the AR of the PAC and combined with the LPE, which realizes the multi-mechanism collaboration enhancement of weak photoacoustic signals. The Allan-Werle deviation evaluation showed that the minimum detection limit of H_2_S in the SF_6_ background is 10.96 ppb when the average time is 200 s. Benefiting from the all-optimization of photoacoustic excitation and detection, the MCEPA has near-field high-sensitivity gas detection capability immune to electromagnetic interference.

## 1 Introduction

Sulfur hexafluoride (SF_6_) is a colorless, odorless industrial synthesis gas with high electrical insulating properties. Electrical equipment with SF_6_ as insulation media, such as gas-insulated switches (GIS) and gas-insulated lines (GIL), have gradually replaced traditional insulation equipment in extra-high and ultra-high voltage power systems [1]. Compared with traditional electrical insulation equipment, SF_6_-based GIS and GIL have outstanding advantages in insulation efficiency and reliability [2]. When the electrical insulation equipment is in a normal state, the chemical property of SF_6_ is very stable. However, in case of overheating or partial discharge of electrical equipment, SF_6_ at the insulation defect will be decomposed into low fluorine compounds (SF_*x*_). The SF_*x*_ reacts with trace amounts of water and oxygen in the equipment. The reaction products H_2_S, SO_2_, CO and other gases will corrode electrical equipment and accelerate the attenuation of insulation performance. Through the real-time measurement and analysis of fault characteristic gas (H_2_S) concentration in electrical equipment, the operation status of GIS and GIL can be effectively monitored and the loss caused by insulation failure can be avoided [3]. In GIS equipment with a normal operating state, the concentration of fault characteristic gas is usually in the order of ppm (H_2_S＜2 ppm, SO_2_＜2 ppm, CO＜10 ppm). For the detection of H_2_S, 1 ppm is usually taken as the warning value. Therefore, the design of a highly sensitive H_2_S analyzer is of great significance for the stable, safe and continuous operation of the power system.

Traditional H_2_S concentration analysis is usually based on the gas chromatograph. The component analysis of the gas chromatograph takes a long time, which can not meet the real-time online monitoring of electrical equipment insulation. Online real-time analysis of H_2_S concentration is usually based on semiconductor sensors and electrochemical sensors. Various metal oxide semiconductors, nanoparticles, and electrolytes are used as sensitive materials for H_2_S [4]. The concentration information of H_2_S can be obtained in real-time by monitoring the change of the electrical characteristics of the sensors. However, semiconductor H_2_S sensors are usually susceptible to cross-interference and the performance of electrochemical gas sensors decays rapidly. Compared with electrical gas sensors, absorption spectroscopy gas analysis technology (ASGAT) has the advantages of online real-time analysis, minor cross-interference between gases, and high detection sensitivity [5], [6], [7]. The ASGAT mainly realizes the detection of gas composition and concentration based on the specificity of the molecules excited by the irradiated beam.

Photoacoustic spectroscopy (PAS) gas detection is an important branch of ASGAT [8], [9], [10]. When the gas to be measured is excited, part of the photons will be absorbed and transition to a high-energy state. The non-radiative transition of gas molecules releases part of the absorbed light energy in the form of heat [11]. When the excitation source is modulated, periodic thermal expansion of the gas produces photoacoustic signals [12], [13]. The concentration of the target gas can be obtained by measuring the photoacoustic signals through acoustic sensors [14], [15], [16]. Benefiting from the background-free detection mechanism, PAS as indirect absorption spectroscopy technology has a wider dynamic range and more sensitive gas detection performance than traditional direct ASGAT. Szabo et al. designed an H_2_S analyzer based on PAS technology. A DFB (central wavelength = 1.57 µm) and an electret acoustic sensor were used for the excitation and acquisition of the H_2_S concentration, respectively. A minimum detection limit (MDL) of 6 ppm was achieved and the detection dynamic range reached four orders of magnitude. However, for the field of power equipment insulation condition monitoring, the detection limit is difficult to meet the application requirements [17]. According to Lambert Beer's law, a higher absorption coefficient will enhance the photoacoustic signals. M. Siciliani et al. achieved highly sensitive detection of H_2_S in the mid-infrared band with a stronger absorption coefficient [18]. When the integration time is 3 s, the detection limit of H_2_S reaches 450 ppb. However, the high cost of quantum cascade laser (EC-QCL) makes it difficult for large-scale applications. S. Viciani. et al. reduced the cost of the mid-infrared H2S detection system by using a DFB laser with a central wavelength of 2.6 µm and the NNEA reached 2.4 × 10^−9^ W cm^−1^ Hz^−1/2^ [19]. However, due to the low power of the excitation light, the detection limit of H_2_S is 4 ppm at the integration time of 1 s. It is difficult to realize real-time and highly sensitive detection of H_2_S in GIS equipment which is usually less than 2 ppm. The excited photoacoustic signals can be enhanced by increasing the excitation power. To improve the detection sensitivity, Wu et al. used an erbium-doped fiber amplifier (EDFA) combined with a near-infrared DFB laser as the excitation light source for H_2_S. A custom-made quartz tuning fork (QTF) with a resonance frequency of 7205 Hz was used as a photoacoustic signal detector [20]. Benefiting from the weak acoustic signal resonance enhancement ability of the QTF, this scheme not only reduces the cost but also maintains highly sensitive gas detection performance [21], [22]. With nitrogen as the background gas, the MDL of H_2_S reaches 890 ppb at 1 s integration time. However, the physical properties such as density and viscosity of SF_6_ are quite different from those of nitrogen. When the background gas is SF_6_, the performance of the H_2_S analyzer will be changed compared with the nitrogen atmosphere. Dong et al. designed an SF_6_ background gas-induced high-Q photoacoustic cell (PAC) with a differential structure [23]. The excitation power of the DFB was amplified to 1.4 W by an EDFA. The highly sensitive detection of H_2_S is achieved through excitation optical power enhancement combined with acoustic resonance amplification. The MDL of 109 ppb was obtained under 1 s integration time. However, the photoacoustic signal sensor based on a condenser microphone or QTF is vulnerable to strong electromagnetic interference around electrical equipment. Electromagnetic interference will affect the measured value during the transmission and conversion of weak electrical signals [24], [25].

In this paper, to realize the real-time and highly sensitive all-optical detection of trace H_2_S in the SF_6_ background, a multi-mechanism collaboration-enhanced photoacoustic analyzer (MCEPA) is proposed. A background gas-induced high-Q resonant PAC is used for acoustic resonance (AR) enhancement of weak photoacoustic signals. A silicon cantilever-based fiber-optic acoustic sensor is used for mechanical resonance (MR) enhancement of photoacoustic signals. Through the optimized design of the H_2_S analyzer, the MR frequency of the fiber-optic silicon cantilever acoustic sensor (FSCAS) is matched with the AR frequency of the PAC. An EDFA was used to cascade the DFB to increase the excitation light power and enhance the photoacoustic signals. Combined with a variety of photoacoustic signal enhancement schemes, the highly sensitive detection of H_2_S is realized. In addition, the MCEPA is immune to electromagnetic interference, which is especially suitable for monitoring the insulation state of electrical equipment.

## 2 Theoretical analysis and optimal design

### 2.1 Design of SF_6_ background gas-induced high-Q PAC

The density, viscosity and sound velocity of SF_6_ are significantly different from those of air or nitrogen. To realize the highly sensitive all-optical photoacoustic detection of trace H_2_S in SF_6_ (background gas), it is necessary to optimize the design of PAC and FSCAS. Photoacoustic gas detection technology is based on the selective interaction between photons and molecules. The specific absorption wavelength and intensity of each substance molecule is the basis for quantitative sensing of gas concentration. The photoacoustic spectroscopy gas detection process mainly includes three stages. First, the target gas molecules absorb excitation light of a specific wavelength and transition to a high-energy state. Second, the absorbed light energy is released through transitions. In the infrared band, at atmospheric pressure, the rate of collisional deactivation is much greater than the rate of radiative decay. Therefore, the light energy is mainly converted into heat energy by molecular collisions and causes the temperature of the gas to increase. Finally, the sound waves generated by the periodic thermal expansion of the gas are sensed by the acoustic sensors. For a target gas with a concentration of *C* and an absorption coefficient of *α*, when the excitation source is *I*(*r*,*t*), the thermal power density source *H*(*r*, *t*) generated in the PAC can be expressed as [26]:

(1) H(r,t)=CαI(r,t)

When the excitation light source is modulated with the angular frequency *ω*, the gas pressure in the PAC periodically fluctuates and photoacoustic signals are generated. The wave equation of sound pressure *p* can be expressed as [27]:

(2) $$\left(\nabla^{2}+\frac{\omega^{2}}{{\upsilon_{s}}^{2}}\right)p\left(r,\omega \right)=\frac{\gamma -1}{{\upsilon_{s}}^{2}}i\omega H\left(r,\omega \right)$$

Where *v*_s_ and *γ* and are the sound speed and heat capacity ratio of the gas in the PAC, respectively. The *p*(*r*, *ω*) is the superposition of multiple modes of sound waves, which can be expanded as:

(3) $$p\left(r,\omega \right)=\sum_{j}A_{j}\left(\omega \right)p_{j}\left(r\right)$$

Where *A*_j_(*ω*) is the amplitude of the sound field. The *p*_j_(*r*) is the acoustic vibration mode, which is determined by the shape of the PAC. When the angular frequency of the photoacoustic signal is equal to the j_th_ order resonance angular frequency (*ω* = *ω*_j_) of the PAC, the sound field amplitude can be expressed as [28]:

(4) $$A_{j}\left(\omega \right)=-\frac{Q_{j}C\alpha }{\omega_{j}}\left[\frac{\gamma -1}{V_{c}}\right]\int p_{j}^{*}\left(r\right)I(r,\omega )dV$$

Where p*_j_(*r*) is the complex conjugate of *p*_j_(*r*), *V*_c_ is the volume of the resonance tube, *ω*_j_ is the *j*_th_-order normal frequency, *Q*_j_ is the acoustic resonance quality factor of the acoustic vibration mode *p*_j_(*r*).

For a cylindrical PAC, when it operates in the first-order longitudinal resonance mode, the sound pressure *A*(*ω*_1_) can be expressed as:

(5) $$A\left(\omega_{1}\right)=P_{0}F_{cell}C\alpha$$

Where *P*_0_ is the excitation light power. The *F*_cell_ is the cell constant, which is used to describe the acoustic cumulative amplification characteristics of the PAC [29]：

(6) $$f_{1}=\frac{v_{s}}{2L_{eff}}$$

(7) $$L_{eff}=L_{PAC}+\frac{16}{3\pi }R_{PAC}$$

(8) $$F_{cell}=-\left(\gamma -1\right)Q_{1}\frac{4L_{eff}^{2}}{V_{c}v_{s}\pi^{2}}$$

Where *f*_1_ and *L*_eff_ are the first-order AR frequency and effective length of the PAC, respectively. The *L*_PAC_ and *R*_PAC_ are the length and radius of the PAC, respectively. When the excitation source is modulated at an eigenfrequency of the resonance tube, the energy from the photoacoustic signal of multiple cycles is accumulated in the standing wave, and the PAC plays the role of acoustic amplification [30]. After the initial transient (energy accumulation in the standing wave), the sound field inside the PAC reaches a steady state. The energy of steady-state sound loss is equal to that of photon absorption. The signal amplification capacity of PAC is determined by the total loss of sound waves. When the resonance tube operates in the first-order longitudinal resonance mode, the *Q*_1_ is used to represent the acoustic amplification performance of the PAC. At atmospheric pressure, the main factors affecting the *Q*_1_ are the viscous gas in the cell and the surface losses caused by heat conduction. The *Q*_1_ can be expressed as [31]：

(9) $$Q_{1}=\frac{R_{PAC}}{\delta_{visc}+\left(\gamma -1\right)\delta_{therm}\left(1+2R_{PAC}/L_{PAC}\right)}$$

Where *δ*_visc_ and *δ*_therm_ are used to describe the thickness of the thermal viscosity boundary layer and thermal boundary layer, respectively:

(10) $$\delta_{visc}=\sqrt{\frac{2\mu }{\omega \rho_{0}}}$$

(11) $$\delta_{therm}=\sqrt{\frac{2kM}{\omega \rho_{0}C_{p}}}$$

Where *ρ*_0,_ *μ*, *k* and *M* are the density, dynamic viscosity, thermal conductivity and molar mass of the gas to be measured, respectively.

**Fig. 1:**
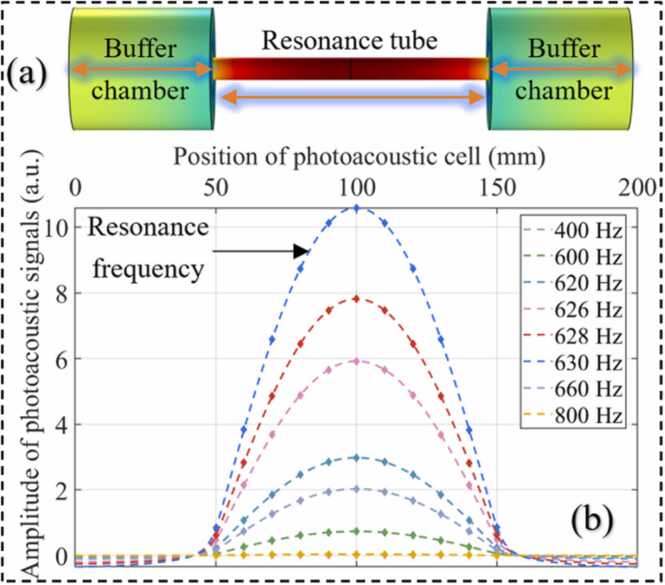
(a) Axial distribution of photoacoustic signals in PAC, (b) the energy accumulation efficiency of sound waves with different frequencies in PAC.

The distribution of the sound field in axial resonant PAC is simulated by the finite element method, as shown in Fig. 1(a). The SF_6_ was used as the background gas in the simulation. The maximum value of the photoacoustic signals is in the middle of the resonance tube. To enhance the sound amplification performance, the structure of the PAC was optimized based on the theoretical analysis results. According to Eq. (4), the photoacoustic signals can be increased by reducing the AR frequency of the PAC. From Eq. (6), the AR frequency can be reduced by increasing the PAC length. Eq. (8) shows that an increase in length will enhance the cell constant of the PAC. However, increasing the length of the PAC will increase the volume of the sensing module, and the consumption of insulating gas inside the electrical equipment will also be increased. According to Eq. (9), increasing the radius of the PAC increases the Q-value. However, as the radius increases, the cell constant decreases. In comprehensive consideration, to obtain a high Q-value and cell constant while maintaining a smaller volume, the designed PAC has a length of 100 mm and a radius of 4 mm. In order to analyze the sound cumulative amplification performance of the PAC, the acoustic field distribution at different frequencies was simulated by COMSOL. As shown in Fig. 1(b), the energy accumulation efficiency of sound waves in PAC will vary with the change of frequency. At the frequency of 630 Hz (AR frequency of the PAC), the enhancement of the sound wave is the largest. Therefore, the maximum sensitivity can be obtained by detecting the photoacoustic signals in the middle of the resonance tube at the laser modulation frequency of 630 Hz.

**Fig. 2:**
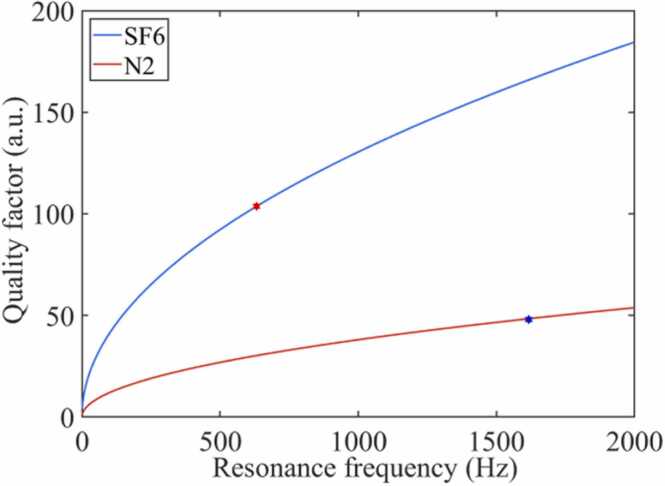
The Q-value of the PAC under different background gases.

Nitrogen or air is usually used as background gas in traditional photoacoustic spectroscopy for trace H_2_S detection. In the field of electrical equipment operating status monitoring, direct measurement of trace fault characteristic components in insulating gas SF_6_ without separation is the basis for real-time online measurement of H_2_S concentration. The acoustic resonance amplification performance of the PAC will be affected by the physical parameters of the background gas. The physical properties of SF_6_ and N_2_ as shown in Table 1. According to (6), (7) and Fig. 1(b), when the background gas is N_2_ and SF_6_, the resonant frequencies of the PAC are 1617 Hz and 630 Hz, respectively. From the Eqs. (9–11) and the Table 1, the quality factor of the PAC change with the resonance frequency is shown in Fig. 2.

**Table 1:** Physical properties of SF_6_ and N_2_.

Gas	*v*_s_ (m/s)	*ρ*_0_ (kg/m^3^)	*γ*	*M* (kg/mol)	*μ* (Pa/s)	*k* W/ (mk)	*C*_p_ (J/molk)
SF_6_	133	6.52	1.1	0.146	1.53 × 10^-5^	0.013	97.5
N_2_	340	1.16	1.4	0.028	1.75 × 10^-5^	0.026	29.1

Analysis results show that the photoacoustic signal generated by the weakly absorbing gas H_2_S can be amplified by the acoustic cumulative effect of the resonant PAC. Compared with N_2_ background, the quality factor of the SF_6_ gas-induced PAC is significantly improved. SF_6_ is used as the background gas to detect the concentration of decomposition product H_2_S, which can avoid the gas separation process and help improve the acoustic amplification performance of the PAC.

### 2.2 Design of fiber-optic cantilever acoustic sensor

According to the sound wave enhancement characteristics of the resonance tube, it is of great significance to design an acoustic sensor with high detection sensitivity at the AR frequency of the PAC. A cantilever diaphragm is a rectangular narrow-band acoustic MR enhancement element with one end clamped and the other end free to vibrate [32], [33]. Compared with the traditional circular acoustic-sensitive diaphragm, the oscillatory of the cantilever is almost not inhibited by the surface tensile stress [34]. Therefore, the sound pressure sensitivity can be improved while maintaining a compact structure [35]. The MR of the cantilever is beneficial to enhance the acoustic sensing ability of the FSCAS. When the photoacoustic signals match the natural frequency of the cantilever, the optimal acoustic sensing performance of the FSCAS can be obtained. The MR frequency can be adjusted by optimizing the three-dimensional dimension. When the cantilever MR matches the AR of the PAC, weak photoacoustic signals will be multi-enhanced.

**Fig. 3:**
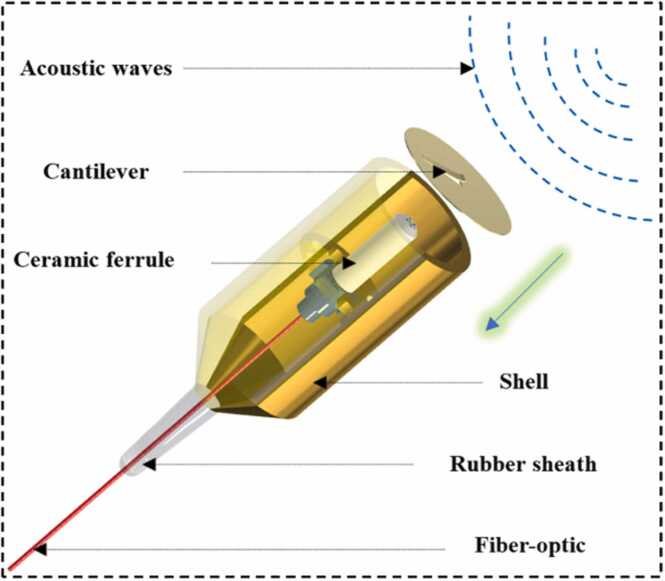
Schematic diagram of FSCAS structure.

The FSCAS based on the Fabry-Perot (F-P) interferometer is composed of a cantilever and a fiber tip as two optical reflecting surfaces combined with a shell. The fiber is fixed by a ceramic ferrule. The structure of FSCAS is shown in Fig. 3. The FSCAS is immune to electromagnetic interference due to components that are all passive. The broad-spectrum light used for cavity-length demodulation is transmitted to the F-P interferometer through single-mode fiber. The cavity length of the F-P interferometer is proportional to the photoacoustic signals. The H_2_S can be measured by demodulating the dynamic variation of the F-P cavity length through a white light interference demodulation algorithm [36], [37].

The performance of the cantilever is crucial for enhancing the gas detection sensitivity. The nanocrystalline silicon-made cantilever has stable properties and excellent sound pressure response characteristics. For a rectangular silicon cantilever, the MR frequency can be expressed as [15], [37], [38]:

(12) $$f_{0}=\frac{t_{c}}{2\pi {L_{c}}^{2}}\sqrt{\frac{2E_{si}}{3\times 0.647\rho_{si}}}$$

Where *E*_si_ and *ρ*_si_ are Young's modulus and density of monocrystalline silicon, respectively. According to Eq. (12), the first-order MR frequency of a cantilever is mainly determined by the length *L*_c_ and the thickness *t*_c_.

**Fig. 4:**
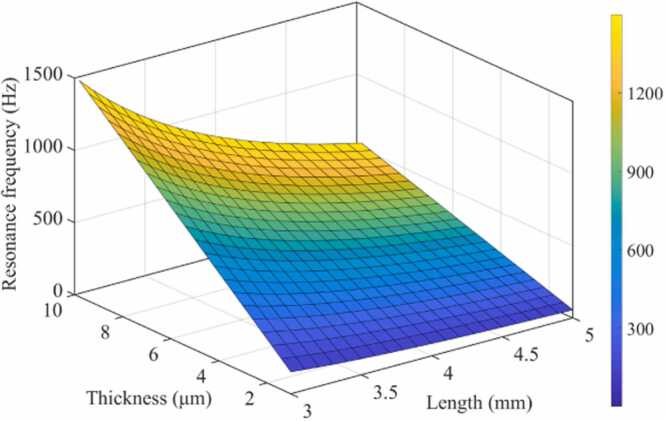
Relationship between MR frequency and size of a silicon cantilever.

**Fig. 5:**
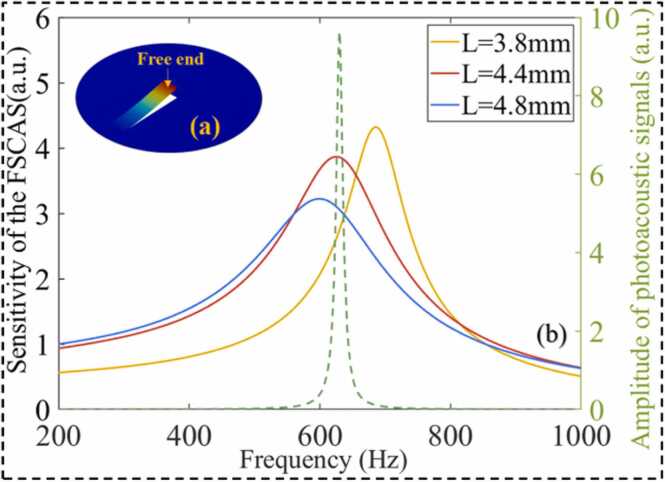
(a) Simulation of the vibration mode, (b) simulation of frequency response curves.

The relationship between the first-order resonant frequency and the size of the cantilever is shown in Fig. 4. To obtain high sound pressure sensitivity while maintaining mechanical strength, the thickness of the cantilever is selected as 6 µm. The effect of air damping of the cantilever with thickness in the micrometer scale cannot be ignored. The amplitude-frequency response of the cantilever under the action of thermal viscous damping was simulated by COMSOL. The result of the vibration mode finite element analysis of the acoustic element is shown in Fig. 5(a). The sound pressure amplitude-frequency response curve of the free end of the cantilever was shown as the solid line in Fig. 5(b). The dotted line is the amplitude-frequency response of the simulated photoacoustic signals in the PAC. When the length, width, and thickness of the cantilever are 4.4 mm, 1 mm, and 6 µm, respectively, the first-order MR frequency of the FSCAS is consistent with the AR enhancement frequency of the PAC. The most sensitive photoacoustic signal sensing performance can be obtained by placing the FSCAS in the middle of the resonance tube.

### 2.3 Excitation light power enhancement

**Fig. 6:**
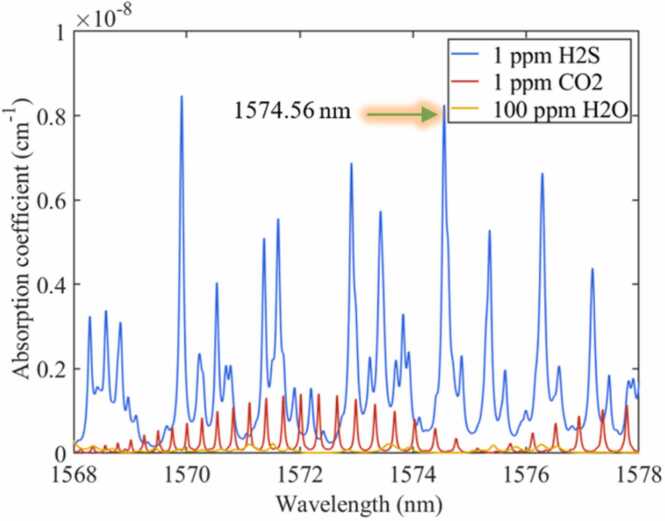
Absorption coefficient of H_2_S, H_2_O and CO_2_ near 1574 nm.

The photoacoustic signals can be enhanced by a stronger gas absorption line and higher optical power absorbed by the H_2_S molecules. The H_2_S has strong absorption bands around 1.6 µm, 2.6 µm and 7.8 µm [39], [40], [41]. The absorption coefficients at 2.6 µm and 7.8 µm are one to two orders of magnitude higher than those at 1.6 µm. However, at 7.8 µm, the detection of H_2_S will be disturbed by the light absorption of background gas SF_6_ molecules. Lasers with a center wavelength of 2.6 µm have low output power and are expensive. In the absorption band of 1.6 µm, the background gas SF_6_ and trace impurity gases (such as H_2_O and CO_2_) that exist in electrical equipment have almost no absorption (about two orders of magnitude lower than H_2_S) at 1574.56 nm and will not interfere with the measurement of H_2_S. In addition, the near-infrared DFB laser has the advantages of high stability, long service life and low price. Therefore, a DFB (central wavelength=1574.56 nm) was used as the excitation laser of H_2_S. The enhancement of the excitation power is realized by cascading the DFB with an EDFA. The multiple enhancement of the photoacoustic signals can be realized by the EDFA-based excitation light power enhancement combined with the background gas-induced high-Q PAC and the narrow-band resonant FSCAS. Fig. 6.

## 3 Experimental results and discussion

**Fig. 7:**
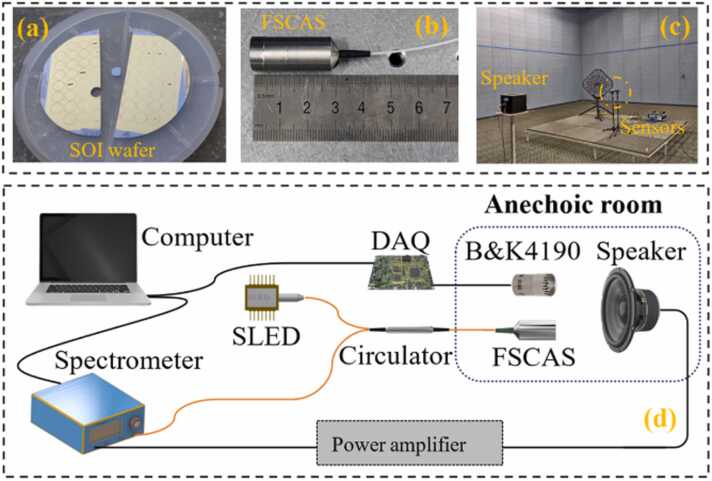
(a) The SOI wafer for fabrication of silicon cantilever diaphragm, (b) the fabricated FSCAS, (c)the test environment of the FSCAS, (d) the diagram of the FSCAS test system.

To achieve multi-mechanism collaboration enhancement of photoacoustic signals, a cantilever with a length of 4.4 mm, a width of 1 mm and a thickness of 6 µm was designed. The dry etching technology process is used to batch fabricate silicon cantilever acoustic wave-sensitive diaphragms on a silicon-on-insulator (SOI) wafer, as shown in Fig. 7(a). The fabricated silicon cantilever diaphragm was fixed to the stainless steel shell with epoxy. The single-mode fiber end was cut flat and held in place by a ceramic ferrule. The completed FSCAS is shown in Fig. 7(b). To verify the acoustic response performance of the sensor, the FSCAS was placed in the full anechoic room (296.15 K, 58%RH, 99.15 kPa), as shown in Fig. 7(c). Fig. 7(d) is the schematic diagram of the FSCAS test system. A SLED with a center wavelength of around 1550 nm was used as a broad-spectrum demodulation light source. The broad-spectrum demodulated beam enters the FSCAS after passing through the fiber circulator. The F-P interference spectrum with cavity length information is formed by two beams of light reflected on the end plane of the fiber and the surface of the cantilever respectively. The high-speed spectrometer receives the interference spectrum and transmits it to the computer. The dynamic cavity length variation Δ*d* is demodulated from the F-P interference spectrum by a white light interference demodulation algorithm [42], [43], [44].

**Fig. 8:**
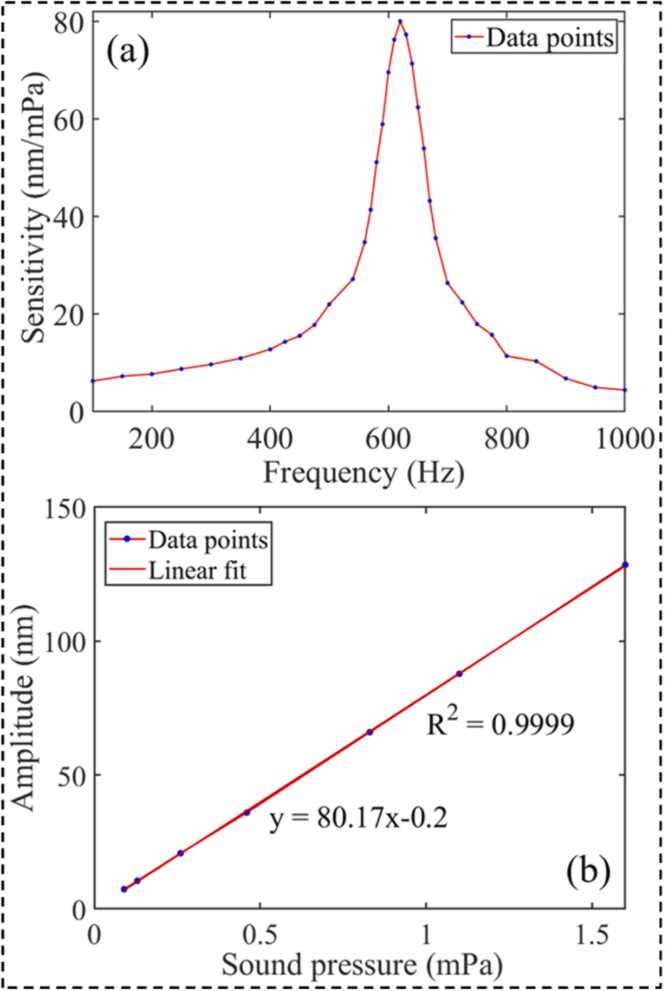
(a) The frequency response curve of the FSCAS, (b) linear fitting of sound pressure response.

To test the sound pressure response characteristics of the FSCAS, the synchronous modulation signal generated by the spectrometer drives the speaker after passing through the power amplifier to emit acoustic waves of different frequencies. The reference microphone unit (B&K 4190) and FSCAS were placed symmetrically on both sides of the acoustic axis. The electrical signal output of the reference microphone unit is collected by the data acquisition (DAQ) module. The sound field information is calculated according to the nominal sensitivity and responsivity of the B&K4190. The sensitivity of FSCAS at different frequencies can be obtained by referring to the sound pressure amplitude monitored by the reference microphone unit combined with the length variation of the F-P cavity. The frequency response curve obtained according to the sensitivity of FSCAS at different frequencies is shown in Fig. 8(a). The experimental results show that the resonance frequency of FSCAS is 620 Hz, which is basically consistent with the simulation results. The FSCAS obtains the best weak acoustic signals sensing ability at 620 Hz, due to the enhanced MR of the cantilever. The vibration amplitude of the cantilever under different sound pressure at the resonance frequency (620 Hz) is collected by changing the loudness of the speaker. The linear fitting result is shown in Fig. 8(b). The FSCAS has good linearity of sound pressure response at 620 Hz, with a sensitivity of 80.17 nm/mPa.

**Fig. 9:**
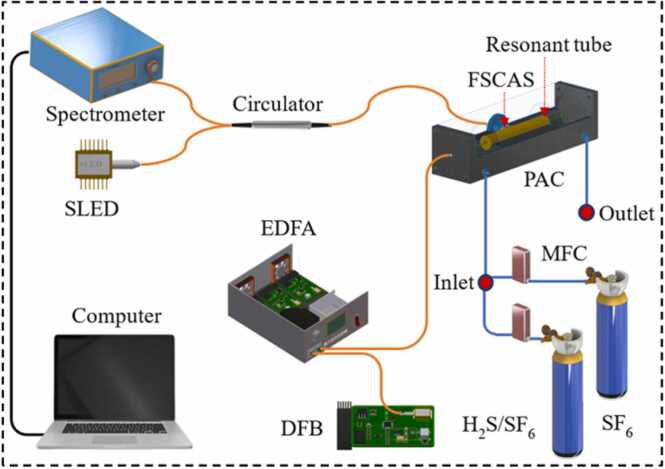
The main components of the MCEPA.

The multi-mechanism collaboration enhanced photoacoustic spectroscopy H_2_S analyzer is shown in Fig. 9. According to the acoustic characteristics of PAC, the AR enhancement efficiency is the highest in the middle of the resonance tube. Therefore, the FSCAS was installed in the middle of the PAC to collect weak photoacoustic signals. The DFB cascaded with an EDFA is used as the excitation light source of H_2_S.

**Fig. 10:**
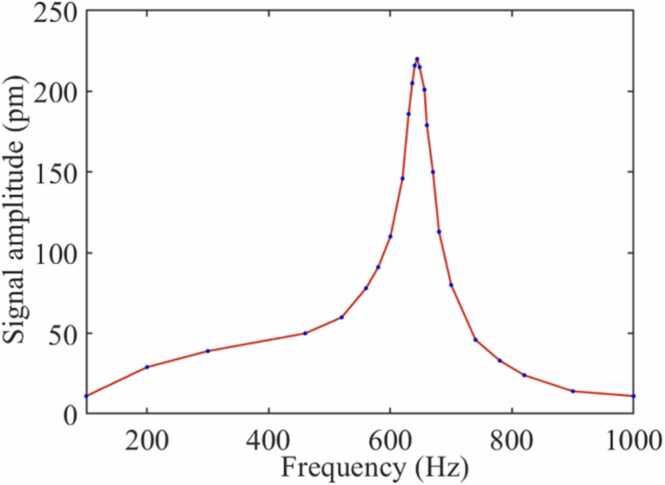
The amplitude-frequency response of the MCEPA.

**Fig. 11:**
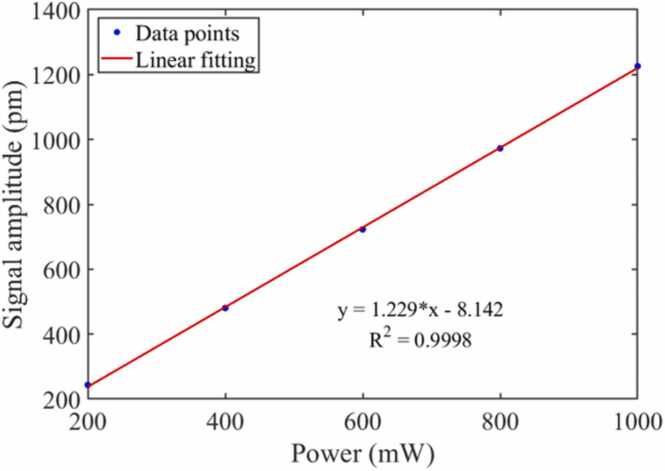
The relationship between the excitation light power and the photoacoustic signal amplitude.

To test the frequency response characteristics of the analyzer, the mixed gas of H_2_S (certified concentration of cylinder: 100.29 ppm) and SF_6_ (certified concentration of cylinder: 99.999%) were filled into the PAC. The volume concentration of H_2_S in the gas to be measured is 100 ppm. The excitation light power output by the EDFA is 200 mW. When the modulation frequency of the DFB is changed, the response of the photoacoustic signals is shown in Fig. 10. According to the unimodal characteristics of amplitude-frequency response, the matching of AR and MR is realized in the working frequency band of the analyzer. The MR of the cantilever and the AR of the PAC were matched at 644 Hz. The power of the excitation light was enhanced by an EDFA. Fig. 11 shows the relationship between the excitation light power and the photoacoustic signal amplitude. With the increase of the excitation light power, the amplitude of the photoacoustic signal is also enhanced. The photoacoustic signals have a good linear relationship with the excitation light power.

**Fig. 12:**
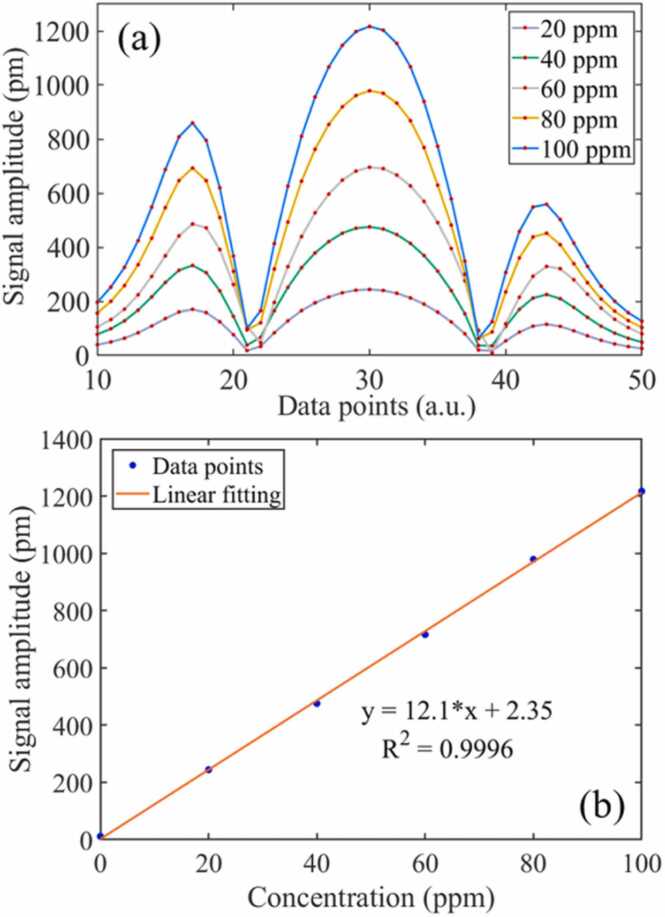
(a) The 2*f*-signals of different concentrations, (b) the linear fitting of the amplitude of 2*f*-signals.

To reduce the interference of cell wall absorption on H_2_S detection, the second harmonic (2 *f*) signal detection technology based on a lock-in amplifier is adopted. The photoacoustic signal reached its peak when the laser modulation frequency was 322 Hz [45]. The modulation frequency of the DFB was fixed at 322 Hz, and the excitation optical power was enhanced to 1000 mW by the EDFA. Different concentrations of H_2_S were mixed by a mass flow controller (MFC) and filled into the PAC. The second harmonic of H_2_S is obtained by modulating the wavelength of the excitation light by superimposing a sawtooth wave with a sine wave. The second harmonic of H_2_S with different concentrations is shown in Fig. 12(a). The integration and acquisition time of each data point is 1 s. The amplitude of the 2*f*-signal was linearly fitted. As shown in Fig. 12(b), the amplitude of the 2*f*-signals has a good linear relationship with the H_2_S concentration. When the excitation light power is 1000 mW, the sensitivity (cantilever vibration amplitude/concentration) of the MCEPA is 12.1 pm/ppm.

**Fig. 13:**
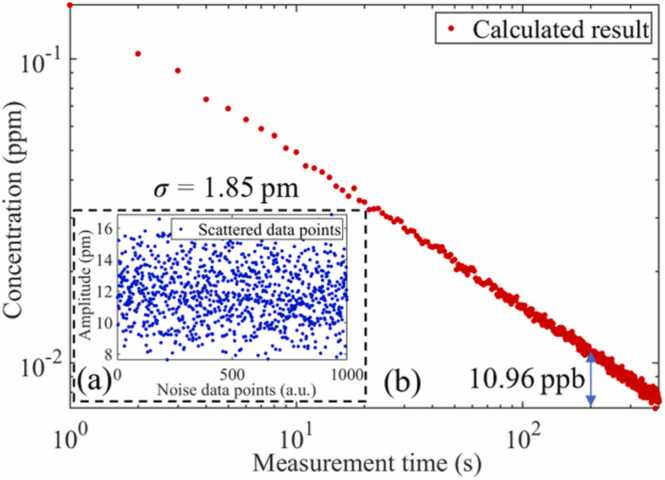
(a) Background noise of the MCEPA, (b) Allan-Werle deviation estimation of the MCEPA.

In order to clarify the detection limit of the analyzer, the background noise was collected and analyzed. High-purity SF_6_ was filled into the PAC and 1000 data were collected continuously. The trace water and CO_2_ molecules in the gas to be measured cannot be completely removed. The absorption of trace impurity molecules and PAC wall results in a non-zero background level. As shown in Fig. 13(a), the standard deviation (1*σ*) of the noise was calculated to be 1.85 pm with an integration time of 1 s, and the noise equivalent MDL of H_2_S under the SF_6_ background is 0.15 ppm. The excitation light power and integration time were normalized, and the NNEA [46], [47] was calculated to be 2.49 × 10^-9^ cm^-1^ W/Hz^1/2^. Allan-Werle deviation [48] was used to evaluate the performance of the H_2_S analyzer. As shown in Fig. 13(b), when the average time is 200 s, the detection limit of H_2_S reaches 10.96 ppb.

The experimental results show that the MCEPA achieves highly sensitive detection of trace H_2_S in SF_6_ due to the collaboration enhancement effect of multiple mechanisms. The background gas-induced high-Q PAC was used for AR enhancement of the photoacoustic signals. The designed and fabricated FSCAS has ultrasensitive acoustic perception performance at the resonant frequency of the PAC. Compared to traditional H_2_S analyzers based on QTFs and condenser microphones, the MCEPA has the advantage of being immune to electromagnetic interference [23], [49], [50], [51], [52]. In addition, the MCEPA achieves or even exceeds the detection performance of traditional H_2_S analyzers in N_2_ background under SF_6_ background gas [49], [50], [51]. The tedious and time-consuming gas separation process is avoided, and real-time high-sensitivity H_2_S detection is realized. Table 2.

**Table 2:** Comparison with other photoacoustic spectroscopy trace H_2_S detectors.

Authors	Detection method/ Background gas	*λ*/Power	*α* (cm^-1^)	NNEA (cm^-1^ W/Hz^1/2^)
Sampaolo et al.[53]	QEPAS/N_2_	104.6 µm/150 mW	3.12	3.1 × 10^−8^
S. Viciani et al.[19]	QEPAS/N_2_	2.6 µm/3 mW	9.25 × 10^−2^	2.4 × 10^−9^
Helman et al.[50]	QEPAS/N_2_	8.1 µm/160 mW	6.70 × 10^−2^	3.05 × 10^−9^
Yin et al.[54]	Condenser microphone/SF_6_	1.58 µm/1.35 W	8.18 × 10^−3^	2.9 × 10^−8^
Dong et al.[55]	Condenser microphone/SF_6_	1.58 µm/1.4 W	8.18 × 10^−3^	2.9 × 10^−9^
This work	F-P interference/SF_6_	1.57 µm/1 W	8.25 × 10^−3^	2.49 × 10^−9^

## 4 Conclusion

In conclusion, a multi-mechanism collaboration enhancement photoacoustic spectroscopy analyzer is proposed for the weakly absorbing gas H_2_S. Theoretical analysis and finite element simulation are used for the fusion and matching of multiple sensitivity enhancement mechanisms. According to the optimized design results, an ultra-sensitive FSCAS and a resonance PAC are fabricated. The performance of FSCAS was tested in an anechoic room, and the acoustic detection sensitivity reaches 80.17 µm/pa. The DFB laser in the near-infrared band cascaded EDFA realizes the enhancement of effective light power absorption. The experimental results of photoacoustic spectroscopy gas analysis show that the MDL of H_2_S is 10.96 ppb when the integration time is 200 s. The MCEPA integrates multiple photoacoustic signal enhancement mechanisms such as the MR of the silicon cantilever, the AR of the resonant PAC, and the LPE of the excitation source. The MCEPA is immune to electromagnetic interference, and all-optical high-sensitivity sensing and determination of trace H_2_S in SF_6_ background gas is realized.

## Funding

This work was supported by the National Nature Science Foundation of China [61905034,62275040]; The Science and Technology Project of State Grid [521205190014]; The Fundamental Research Funds for the Central Universities [DUT21JC03].

## Declaration of Competing Interest

The authors declare no conflicts of interest.

## Data Availability

The authors are unable or have chosen not to specify which data has been used.

## References

[1] Hong-Yang Z., Guo-Ming M., Wang Y., Wei-Qi Q., Jiang J., Yan C., Cheng-Rong L. (2019). Optical sensing in condition monitoring of gas insulated apparatus: a review. High Volt..

[2] Yin X., Wu H., Dong L., Ma W., Zhang L., Yin W., Xiao L., Jia S., Tittel F.K. (2019). Ppb-level photoacoustic sensor system for saturation-free CO detection of SF6 decomposition by use of a 10 W fiber-amplified near-infrared diode laser. Sens. Actuators B Chem..

[3] Yin X., Dong L., Wu H., Zhang L., Ma W., Yin W., Xiao L., Jia S., Tittel F.K. (2019). Highly sensitive photoacoustic multicomponent gas sensor for SF 6 decomposition online monitoring. Opt. Express.

[4] Verma M.K., Gupta V. (2012). A highly sensitive SnO 2-CuO multilayered sensor structure for detection of H 2S gas. Sens. Actuators B Chem..

[5] Xu L., Zhou S., Liu N., Zhang M., Liang J., Li J. (2020). Multigas sensing technique based on quartz crystal tuning fork-enhanced laser spectroscopy. Anal. Chem..

[6] Liu N., Xu L., Zhou S., Zhang L., Li J. (2020). Simultaneous detection of multiple atmospheric components using an NIR and MIR laser hybrid gas sensing system. ACS Sens..

[7] Ma Y., Hu Y., Qiao S., Lang Z., Liu X., He Y., Spagnolo V. (2022). Quartz tuning forks resonance frequency matching for laser spectroscopy sensing. Photoacoustics.

[8] Huang Q., Wei Y., Li J. (2022). Simultaneous detection of multiple gases using multi-resonance photoacoustic spectroscopy. Sens. Actuators B Chem..

[9] Chen K., Deng H., Guo M., Luo C., Liu S., Zhang B., Ma F., Zhu F., Gong Z., Peng W., Yu Q. (2020). Tube-cantilever double resonance enhanced fiber-optic photoacoustic spectrometer. Opt. Laser Technol..

[10] Zhang G., Chen K., Guo M., Li C., Xu L., Wang N., Zhao X. (2022). Miniature 3D-printed resonant photoacoustic cell for flowing gas detection. Sens. Actuators A Phys..

[11] Zhao X., Chen K., Cui D., Guo M., Li C., Qi H., Zhang G., Gong Z., Zhou Z., Peng W. (2022). Ultra-high sensitive photoacoustic gas detector based on differential multi-pass cell. Sens. Actuators B Chem..

[12] Qiao S., Ma Y., Patimisco P., Sampaolo A., He Y., Lang Z., Tittel F.K., Spagnolo V. (2021). Multi-pass quartz-enhanced photoacoustic spectroscopy-based trace gas sensing. Opt. Lett..

[13] Guo M., Chen K., Yang B., Zhang G., Zhao X., Li C. (2022). Miniaturized anti-interference cantilever-enhanced fiber-optic photoacoustic methane sensor. Sens. Actuators B Chem..

[14] Zhang X., Cheng Z., Li X. (2016). Cantilever enhanced photoacoustic spectrometry: quantitative analysis of the trace H2S produced by SF6 decomposition. Infrared Phys. Technol..

[15] Guo M., Chen K., Li C., Xu L., Zhang G., Wang N., Li C., Ma F., Gong Z., Yu Q. (2022). High-Sensitivity Silicon Cantilever-Enhanced Photoacoustic Spectroscopy Analyzer with Low Gas Consumption. Anal. Chem..

[16] Li C., Chen K., Zhao J., Qi H., Zhao X., Ma F., Han X., Guo M., An R. (2023). High-sensitivity dynamic analysis of dissolved gas in oil based on differential photoacoustic cell. Opt. Lasers Eng..

[17] Szabó A., Mohácsi Á., Gulyás G., Bozóki Z., Szabó G. (2013). In situ and wide range quantification of hydrogen sulfide in industrial gases by means of photoacoustic spectroscopy. Meas. Sci. Technol..

[18] Siciliani de Cumis M., Viciani S., Borri S., Patimisco P., Sampaolo A., Scamarcio G., De Natale P., D’Amato F., Spagnolo V. (2014). Widely-tunable mid-infrared fiber-coupled quartz-enhanced photoacoustic sensor for environmental monitoring. Opt. Express.

[19] Viciani S., Siciliani de Cumis M., Borri S., Patimisco P., Sampaolo A., Scamarcio G., De Natale P., D’Amato F., Spagnolo V. (2015). A quartz-enhanced photoacoustic sensor for H2S trace-gas detection at 2.6 μm. Appl. Phys. B.

[20] Wu H., Sampaolo A., Dong L., Patimisco P., Liu X., Zheng H., Yin X., Ma W., Zhang L., Yin W., Spagnolo V., Jia S., Tittel F.K. (2015). Quartz enhanced photoacoustic H2S gas sensor based on a fiber-amplifier source and a custom tuning fork with large prong spacing. Appl. Phys. Lett..

[21] Shang Z., Li S., Li B., Wu H., Sampaolo A., Patimisco P., Spagnolo V., Dong L. (2022). Quartz-enhanced photoacoustic NH3 sensor exploiting a large-prong-spacing quartz tuning fork and an optical fiber amplifier for biomedical applications. Photoacoustics.

[22] Sgobba F., Sampaolo A., Patimisco P., Giglio M., Menduni G., Ranieri A.C., Hoelzl C., Rossmadl H., Brehm C., Mackowiak V., Assante D., Ranieri E., Spagnolo V. (2022). Compact and portable quartz-enhanced photoacoustic spectroscopy sensor for carbon monoxide environmental monitoring in urban areas. Photoacoustics.

[23] Yin X., Dong L., Wu H., Ma W., Zhang L., Yin W., Xiao L., Jia S., Tittel F.K. (2017). Ppb-level H2S detection for SF6 decomposition based on a fiber-amplified telecommunication diode laser and a background-gas-induced high- Q photoacoustic cell. Appl. Phys. Lett..

[24] Y. Zhang, K. Rasmussen Detect. Electromagn. Interf. Attacks Sens. Syst. 2020 203 216 doi: 10.1109/SP40000.2020.00001.

[25] C. Tsai, C. Fann, S. Wang, R. Fung, Paramagn. Oxyg. Meas. Using Opt. -Fiber Micro, 73, 2001, pp. 211–215.

[26] Yang T., Chen W., Wang P. (2021). A review of all-optical photoacoustic spectroscopy as a gas sensing method. Appl. Spectrosc. Rev..

[27] Gong Z., Chen K., Chen Y., Mei L., Yu Q. (2019). Integration of T-type half-open photoacoustic cell and fiber-optic acoustic sensor for trace gas detection. Opt. Express.

[28] Chen K., Chen Y., Zhang B., Mei L., Guo M., Deng H., Liu S., Ma F., Gong Z., Yu Q. (2020). Highly sensitive photoacoustic microcavity gas sensor for leak detection. Sensors.

[29] Mohebbifar M.R. (2019). High-sensitivity detection and quantification of CHCl3 vapors in various gas environments based on the photoacoustic spectroscopy. Microw. Opt. Technol. Lett..

[30] Gong Z., Gao T., Mei L., Chen K., Chen Y., Zhang B., Peng W., Yu Q. (2021). Ppb-level detection of methane based on an optimized T-type photoacoustic cell and a NIR diode laser. Photoacoustics.

[31] Besson J.P., Schilt S., Thévenaz L. (2004). Multi-gas sensing based on photoacoustic spectroscopy using tunable laser diodes. Spectrochim. Acta A Mol. Biomol. Spectrosc..

[32] Wei W., Zhu Y., Lin C., Tian L., Xu Z., Nong J. (2015). All-optical cantilever-enhanced photoacoustic spectroscopy in the open environment. Int. J. Thermophys..

[33] Liu K., Cao Y., Wang G., Zhang W., Chen W., Gao X. (2018). A novel photoacoustic spectroscopy gas sensor using a low cost polyvinylidene fluoride film. Sens Actuators B Chem..

[34] Chen K., Guo M., Liu S., Zhang B., Deng H., Zheng Y., Chen Y., Luo C., Tao L., Lou M., Yu Q. (2019). Fiber-optic photoacoustic sensor for remote monitoring of gas micro-leakage. Opt. Express.

[35] Chen K., Guo M., Yang B., Jin F., Wang G., Ma F., Li C., Zhang B., Deng H., Gong Z. (2021). Highly sensitive optical fiber photoacoustic sensor for in situ detection of dissolved gas in oil. IEEE Trans. Instrum. Meas..

[36] Chen K., Zhang B., Guo M., Chen Y., Deng H., Yang B., Liu S., Ma F., Zhu F., Gong Z., Yu Q. (2020). Photoacoustic trace gas detection of ethylene in high-concentration methane background based on dual light sources and fiber-optic microphone. Sens. Actuators B Chem..

[37] Chen K., Yang B., Guo M., Deng H., Zhang B., Liu S., Li C., An R., Peng W., Yu Q. (2020). Fiber-optic photoacoustic gas sensor with temperature self-compensation. Opt. Lett..

[38] Wu G., Gong Z., Ma J., Li H., Guo M., Chen K., Peng W., Yu Q., Mei L. (2022). High-sensitivity miniature dual-resonance photoacoustic sensor based on silicon cantilever beam for trace gas sensing. Photoacoustics.

[39] Varga A., Bozóki Z., Szakáll M., Szabó G. (2006). Photoacoustic system for on-line process monitoring of hydrogen sulfide (H2S) concentration in natural gas streams. Appl. Phys. B.

[40] Nikodem M. (2016). Chirped laser dispersion spectroscopy for laser-based hydrogen sulfide detection in open-path conditions. Opt. Express.

[41] Viciani S., Siciliani de Cumis M., Borri S., Patimisco P., Sampaolo A., Scamarcio G., De Natale P., D’Amato F., Spagnolo V. (2015). A quartz-enhanced photoacoustic sensor for H2S trace-gas detection at 2.6 μm. Appl. Phys. B.

[42] Guo M., Chen K., Yang B., Li C., Zhang B., Yang Y., Wang Y., Li C., Gong Z., Ma F., Yu Q. (2021). Ultrahigh sensitivity fiber-optic fabry-perot interferometric acoustic sensor based on silicon cantilever. IEEE Trans. Instrum. Meas..

[43] Chen K., Yu Z., Yu Q., Guo M., Zhao Z., Qu C., Gong Z., Yang Y. (2018). Fast demodulated white-light interferometry-based fiber-optic Fabry–Perot cantilever microphone. Opt. Lett..

[44] Ke Chen, Zhihao Yu, Zhenfeng Gong, Lock- White-Light-Interferom. -Based all-Opt. Photoacoust. spectrometer 43 2018 5038 5041 doi: 10.1364/OL.43.005038. 10.1364/OL.43.005038 30320813

[45] Ma Y., He Y., Zhang L., Yu X., Zhang J., Sun R., Tittel F.K. (2017). Ultra-high sensitive acetylene detection using quartz-enhanced photoacoustic spectroscopy with a fiber amplified diode laser and a 30.72 kHz quartz tuning fork. Appl. Phys. Lett..

[46] Wu H., Dong L., Zheng H., Yu Y., Ma W., Zhang L., Yin W., Xiao L., Jia S., Tittel F.K. (2017). Beat frequency quartz-enhanced photoacoustic spectroscopy for fast and calibration-free continuous trace-gas monitoring. Nat. Commun..

[47] Li S., Dong L., Wu H., Sampaolo A., Patimisco P., Spagnolo V., Tittel F.K. (2019). Ppb-level quartz-enhanced photoacoustic detection of carbon monoxide exploiting a surface grooved tuning fork. Anal. Chem..

[48] Wu H., Dong L., Zheng H., Liu X., Yin X., Ma W., Zhang L., Yin W., Jia S., Tittel F.K. (2015). Enhanced near-infrared QEPAS sensor for sub-ppm level H2S detection by means of a fiber amplified 1582 nm DFB laser. Sens Actuators B Chem..

[49] Sampaolo A., Yu C., Wei T., Zifarelli A., Giglio M., Patimisco P., Zhu H., Zhu H., He L., Wu H., Dong L., Xu G., Spagnolo V. (2021). H2S quartz-enhanced photoacoustic spectroscopy sensor employing a liquid-nitrogen-cooled THz quantum cascade laser operating in pulsed mode. Photoacoustics.

[50] Helman M., Moser H., Dudkowiak A., Lendl B. (2017). Off-beam quartz-enhanced photoacoustic spectroscopy-based sensor for hydrogen sulfide trace gas detection using a mode-hop-free external cavity quantum cascade laser. Appl. Phys. B.

[51] F. Zhao, Y. Gao, L. Yang, Y. Yan, J. Li, J. Ren, S. Russo, A. Zifarelli, P. Patimisco, H. Wu, applied sciences Near-Infrared Quartz-Enhanced Photoacoustic Sensor for H 2 S Detection in Biogas, (n.d.).

[52] Yin X., Su Y., Xi T., Chen B., Zhang L., Zhang X., Liu L., Shao X. (2022). Research progress on photoacoustic SF6decomposition gas sensor in gas-insulated switchgear. J. Appl. Phys..

[53] Sampaolo A., Yu C., Wei T., Zifarelli A., Giglio M., Patimisco P., Zhu H., Zhu H., He L., Wu H., Dong L., Xu G., Spagnolo V. (2021). H2S quartz-enhanced photoacoustic spectroscopy sensor employing a liquid-nitrogen-cooled THz quantum cascade laser operating in pulsed mode. Photoacoustics.

[54] Yin X., Dong L., Wu H., Zhang L., Ma W., Yin W., Xiao L., Jia S., Tittel F.K. (2019). Highly sensitive photoacoustic multicomponent gas sensor for SF 6 decomposition online monitoring. Opt. Express.

[55] Yin X., Dong L., Wu H., Ma W., Zhang L., Yin W., Xiao L., Jia S., Tittel F.K. (2017). Ppb-level H2S detection for SF6 decomposition based on a fiber-amplified telecommunication diode laser and a background-gas-induced high- Q photoacoustic cell. Appl. Phys. Lett..

